# Analysis of a Urinary Biomarker Panel for Clinical Outcomes Assessment in Cirrhosis

**DOI:** 10.1371/journal.pone.0128145

**Published:** 2015-06-04

**Authors:** Xavier Ariza, Elsa Solà, Chiara Elia, Rogelio Barreto, Rebeca Moreira, Manuel Morales-Ruiz, Isabel Graupera, Ezequiel Rodríguez, Patricia Huelin, Cristina Solé, Javier Fernández, Wladimiro Jiménez, Vicente Arroyo, Pere Ginès

**Affiliations:** 1 Liver Unit, Hospital Clínic, University of Barcelona, Barcelona, Catalunya, Spain; 2 Institut d’Investigacions Biomèdiques August-Pi-Sunyer (IDIBAPS), Barcelona, Spain; 3 Centro de Investigación Biomédica en Red de Enfermedades Hepáticas y Digestivas (CIBEREHED), Barcelona, Spain; 4 Instituto Reina Sofía de Investigación Nefrológica (IRSIN), Barcelona, Spain; 5 Biochemistry and Molecular Genetics Department, Hospital Clínic, University of Barcelona, Barcelona, Catalunya, Spain; University of Torino, ITALY

## Abstract

**Background:**

Biomarkers are potentially useful in assessment of outcomes in patients with cirrhosis, but information is very limited. Given the large number of biomarkers, adequate choice of which biomarker(s) to investigate first is important.

**Aim:**

Analysis of potential usefulness of a panel of urinary biomarkers in outcome assessment in cirrhosis.

**Patients and Methods:**

Fifty-five patients with acute decompensation of cirrhosis were studied: 39 had Acute Kidney Injury (AKI) (Prerenal 12, type-1 HRS (hepatorenal syndrome) 15 and Acute Tubular Necrosis (ATN) 12) and 16 acute decompensation without AKI. Thirty-four patients had Acute-on-chronic liver failure (ACLF). A panel of 12 urinary biomarkers was assessed, using a multiplex assay, for their relationship with ATN, ACLF and mortality.

**Results:**

Biomarker with best accuracy for ATN diagnosis was NGAL (neutrophil-gelatinase associated lipocalin): 36 [26-125], 104 [58-208] and 1807 [494-3,716] μg/g creatinine in Prerenal-AKI, type-1 HRS and ATN, respectively; p<0.0001 (AUROC 0.957). Other attractive biomarkers for ATN diagnosis were IL-18, albumin, trefoil-factor-3 (TFF-3) and glutathione-S-transferase-π (GST-π) Biomarkers with less accuracy for ATN AUCROC<0.8 were β2-microglobulin, calbindin, cystatin-C, clusterin and KIM-1 (kidney injury molecule-1). For ACLF, the biomarker with the best accuracy was NGAL (ACLF vs. No-ACLF: 165 [67-676] and 32 [19-40] μg/g creatinine; respectively; p<0.0001; AUROC 0.878). Interestingly, other biomarkers with high accuracy for ACLF were osteopontin, albumin, and TFF-3. Biomarkers with best accuracy for prognosis were those associated with ACLF.

**Conclusions:**

A number of biomarkers appear promising for differential diagnosis between ATN and other types of AKI. The most interesting biomarkers for ACLF and prognosis are NGAL, osteopontin, albumin, and TFF-3. These results support the role of major inflammatory reaction in the pathogenesis of ACLF.

## Introduction

Biomarkers are important to help in decision making and outcome prediction of chronic conditions. For many years, clinical decisions in patients with cirrhosis have relied on the use of simple liver function tests, either used alone or in combination [1,2]. Moreover, because cirrhosis is frequently associated with failure of extrahepatic organs, a condition known as Acute-on-chronic liver failure (ACLF), biomarkers of function of organs other than the liver may also be of major importance in therapeutic decisions and prediction of prognosis [3,4]. In this regard, serum creatinine has been extensively used in the assessment of kidney dysfunction associated with cirrhosis despite its limitations as an estimate of glomerular filtration rate (GFR) [5]. Nevertheless, serum creatinine is not able to distinguish between the different causes of kidney dysfunction that can affect patients with cirrhosis. Therefore, urinary biomarkers are obviously needed in the differential diagnosis of kidney failure, particularly because some of the causes have specific management and there are no objective parameters useful in this context. Finally, since cirrhosis is frequently associated with a proinflammatory state, biomarkers of systemic inflammation should also be explored. Overall, there is a need for new and accurate biomarkers in the clinical assessment of patients with cirrhosis.

The discovery of biomarkers has advanced rapidly in the field of kidney diseases because of the easy access to the analysis of fluid that is coming out directly from the kidney [6,7]. By contrast, the investigation of biomarkers from the liver is going at a much slower pace because of the difficulty in accessing the fluid output from the liver, among other potential reasons. Nonetheless, some markers derived from non-renal origin may appear in the urine through glomerular filtration. For example, several studies have convincingly shown that neutrophil-gelatinase associated lipocalin (NGAL) in addition of being synthetized in the kidney under conditions of tubular injury is synthetized in the liver as a result of liver cell injury or liver regeneration [8–10]. Therefore, this protein may be a potential biomarker of liver injury.

There are only a handful of studies evaluating urinary biomarkers in patients with liver diseases, particularly cirrhosis [11–15]. These studies have investigated either a single biomarker or a small number of biomarkers. Therefore, the potential usefulness of some biomarkers has not been investigated yet. Moreover, the existing information in cirrhosis is exclusively focused on the assessment of biomarkers in the differential diagnosis of the cause of Acute Kidney Injury (AKI), but their possible role in the evaluation of other outcomes has not been assessed. In this context, we evaluated a panel of 12 biomarkers identified in animal studies of AKI. The panel includes the most commonly investigated biomarkers in the field of AKI (NGAL, kidney injury molecule-1—KIM-1—and interleukin-18—IL-18–). Moreover, the panel has the six biomarkers recently proposed by the Food and Drug Administration and the European Medicines Evaluation Agency for use as indicators of nephrotoxic AKI through the Critical Path Initiave (albumin, β2-microglobulin, clusterin, cystatin C, KIM-1 and trefoil factor-3-TFF-3-) [16]. Finally, the panel also includes some proinflammatory molecules, such as monocyte chemoattractant protein-1 (MCP-1) and osteopontin, together with calbindin and glutathione-S-transferase-π (GST-π). In addition of investigating the role of all these biomarkers in the evaluation of AKI, their possible role in the assessment of ACLF as well as prognosis has been evaluated.

## Patients and Methods

### Patient population and study design

This study was presented and approved by the Ethics Committee of Hospital Clinic registration number 2012/7646. The creation of the biobank collection was presented and approved by the Ethics Committee registration number 2011/6689. All patient samples were included and stored in the Biobank as required by Spanish Law. All patients signed a written informed consent document and gave permission for samples to be used in the study following current national and institutional guidelines for sample storage and usage for research purposes.

Fifty-five patients admitted to the Liver Unit of the Hospital Clínic of Barcelona for the management of an acute decompensation of cirrhosis were included in the study. These patients were selected from a prospective database which includes all consecutive patients with cirrhosis admitted to hospital for an acute decompensation of the disease since December 2011. The only exclusion criteria are: 1) chronic hemodialysis before admission; (2) previous liver and/or kidney transplantation; (3) hepatocellular carcinoma outside the Milan criteria or any other advanced malignancy; and (4) lack of informed consent. Because the study was an exploratory analysis of the relationship between urinary biomarkers and clinical outcomes, and AKI was the primary outcome, we selected from the database a sizeable number of consecutive patients with AKI due to three different causes, prerenal failure, type-1 hepatorenal syndrome (HRS) and acute tubular necrosis (ATN), as well as patients with an acute decompensation of cirrhosis who neither had AKI at admission nor developed it during hospitalization. A total of 55 patients were included, 39 with AKI and 16 with acute decompensation of cirrhosis without AKI. A group of 6 healthy volunteers was also studied. In patients with AKI the collection of urine was performed at the time of diagnosis of AKI (median time between diagnosis and urine collection was 1 day—range 0 to 2 days). In patients without AKI, urine was collected at the time of admission to hospital. Samples were centrifuged at 1,000 rpm for 10 min and the supernatant stored in cryovials at -80°C until analysis. All clinical information of patients including baseline demographic, clinical, and biochemical variables and outcomes during hospitalization was collected prospectively using specifically designed electronic case report forms.

### Clinical outcomes and definitions

AKI was defined using the AKIN criteria, evaluated in intervals of 48–72 h throughout hospitalization, as an increase in serum creatinine of ≥0.3 mg/dl or ≥50% over the baseline value obtained in the previous 48–72 h [17]. For the diagnosis of AKI at admission the value used as baseline was the most recent stable serum creatinine value available within the previous 3 months [13,15].

Prerenal-AKI, type-1 HRS, and ATN were defined as previously reported [11,18]. Treatment of different types of AKI was performed according to international guidelines [19,20]. Definition and grading of ACLF was done according to the CANONIC study [4]. Survival was evaluated at 3 months.

### Measurement of urinary biomarkers

Bio-Plex Pro RBM Human Kidney Toxicity panel 1 (calbindin, clusterin, GST-π, IL-18, KIM-1, and MCP-1) and panel 2 (albumin, β2-microglobulin, cystatin C, NGAL, osteopontin, and TFF-3) were used according to the manufacturer protocol (Bio-Rad Laboratories, Hercules, CA). Briefly, urine samples were centrifuged at 500 x g for 5 min and diluted 1:4 for the panel 1 and 1:50 for the panel 2 assays. After blockade of nonspecific binding sites, 30 microliters of standards, controls or diluted samples were added to 96-well plates and incubated with 10 mL of fluorescently dyed magnetic microspheres covalently coupled to specific antibodies for the desired biomarkers. Next, the plates were incubated for 1 h at room temperature and washed three times with wash buffer. Afterwards, 40 mL of biotinylated detection antibody was added to the wells and the plates were incubated for an additional hour at room temperature. The final detection complex was formed with the addition of streptavidin-phycoerythrin conjugated. The median relative fluorescence units from the antibody reactions were acquired using a Luminex 200 analyzer (Luminex, Austin, TX, USA) and the Bio-Plex Manager Software (v. 6.0; Bio-Rad Laboratories, Hercules, CA). The urinary biomarkers were indexed to urinary creatinine for all analyses to adjust for variability in urine concentration. Since some values of albumin concentration obtained with the multiplex assay were above the upper limit of detection, all albumin measurements were repeated using the ADVIA Chemistry Microalbumin_2 immunoturbidimetry method, enhanced with polyethylene glycol, (Siemens Healthcare Diagnostics, Deerfield, IL).

### Statistical analysis

Comparisons of continuous variables between groups were made with ANOVA or Kruskal-Wallis, while comparison between specific groups was made with post-tests. Categorical variables were compared with chi-square test. The area under the receiver-operating characteristic curves (AUCROC) was used to assess the relationship between each biomarker and every one of the specific outcomes. To assess the relationship between biomarkers and ATN, patients with AKI were divided into those with ATN and those without ATN (i.e Prerenal plus type-1 HRS). All statistical analyses were performed using SPSS 20.0 software. Biomarker values are expressed as median and interquartile ranges. The significance level for all statistical tests was set at 0.05 two-tailed.

## Results

### Baseline characteristics of patients

The baseline characteristics of patients included in the study are shown in Table 1. Most patients had advanced cirrhosis as indicated by markedly abnormal liver function tests and high Child-Pugh and MELD scores together with impairment of kidney function. Moreover, 62% of patients fulfilled the criteria of ACLF at the time of inclusion in the study.

**Table 1:** Demographic, clinical data and liver and kidney function of all patients included.

Age, yr	58 ± 10
Male, n (%)	43 (78%)
Chronic impairment of kidney function, n (%)*	3 (5.5%)
Etiology of cirrhosis, n: Alcoholic /hepatitis C/ other	26/19/10
Norfloxacin prophylaxis	10 (18%)
Treatment with beta-blockers	20 (36%)
Presence of ascites	46 (84%)
Presence of encephalopathy	18 (33%)
Presence of bacterial infection	28 (51%)
Presence of shock	12 (22%)
Serum bilirubin (mg/dL)	6.6 ± 10.5
Serum albumin (g/L)	29.0 ± 6.1
INR	1.8 ± 0.6
MELD score	23 ± 9
Child-Pugh score	9.2 ± 1.9
ACLF**	34 (62%)
Serum creatinine (mg/dL)	2.3 ± 1.6
Serum sodium (mEq/L)	132 ± 6
Mean arterial pressure (mmHg)	76 ± 13
Blood leukocytes (x10^9^/L)	8.3 ± 5.6
C-reactive protein (mg/dL)	4.3 ± 3.7

Values are mean ± SD or number and percentages.

* Parenchymal nephropathy in 2 patients and type-2 hepatorenal in 1 patient.

INR, international normalized ratio; MELD, model for end-stage liver disease.

**ACLF, Acute-on-chronic liver failure, ACLF grade I in 19 patients, grade II in 7 patients and grade III in 8 patients.

Thirty-nine of the 55 (71%) patients had AKI. AKI was stage 1 in 13 patients (AKI-1A in one patient and AKI-1B in 12 patients) [18,21], stage 2 in 15, and stage 3 in 11 patients. The comparison of demographic, clinical, and laboratory variables between patients with and without AKI is shown in Table A in S1 File. The type of AKI was Prerenal in 12 patients, type-1 HRS in 15 patients, and ATN in the remaining 12 patients. Table 2 shows the characteristics of patients categorized according to the presence and type of AKI. Patients with ATN and those with type-1 HRS had more marked impairment of liver, kidney, and circulatory function compared to patients with Prerenal-AKI or no AKI. Among the four groups of patients, the greatest impairment of liver and kidney function tests and systemic hemodynamics was observed in patients with ATN.

**Table 2:** Characteristics of patients according to the presence and type of AKI.

	No AKI (n = 16)	Prerenal (n = 12)	HRS Type 1 (n = 15)	ATN (n = 12)	*P*
Age, yr	58 ± 10	63 ± 6	59 ± 11	50 ± 9	**0.006**
Male, n (%)	13 (81%)	9 (75%)	14 (93%)	7 (58%)	0.176
Alcoholic cirrhosis	6 (38%)	7 (58%)	8 (53%)	5 (42%)	0.275
Presence of ascites	12 (75%)	9 (75%)	15 (100%)	10 (83%)	0.216
Presence of encephalopathy	3 (19%)	2 (17%)	5 (33%)	8 (67%)	**0.028**
Presence of bacterial infection	8 (50%)	0 (0%)	9 (60%)	11 (92%)	**< 0.0001**
Serum bilirubin (mg/dL)	1.9 ± 0.9	1.8 ± 1.1	5.5 ± 7.4	19.3 ± 15.1	**< 0.0001**
INR	1.5 ± 0.4	1.5 ± 0.3	1.9 ± 0.6	2.1 ± 0.9	**0.019**
MELD score	13 ± 4	20 ± 5	29 ± 5	31 ± 7	**< 0.0001**
Child-Pugh score	8.6 ± 1.5	8.3 ± 1.9	9.9 ± 1.9	10.3 ± 1.6	**0.012**
Serum creatinine (mg/dL)	0.7 ± 0.2	2.3 ± 1.1	3.1 ± 0.8	3.3 ± 2.2	**< 0.0001**
Serum sodium (mEq/L)	135 ± 5	133 ± 6	129 ± 7	131 ± 6	0.075
Mean arterial pressure (mmHg)	84 ± 14	76 ± 14	73 ± 9	68 ± 9	**0.005**
Blood leukocytes (x10^9^/L)	5.9 ± 3.1	6.6 ± 3.6	7.3 ± 5.1	14.1 ± 6.8	**< 0.0001**
C-reactive protein (mg/dL)	3.2 ± 3.3	3.3 ± 4.6	6.7 ± 4.2	3.7 ± 2.1	0.094
Urine sodium (mEq/L)	46 ± 43	31 ± 22	14 ± 23	32 ± 31	0.062
FENa (%)	0.3 ± 0.4	0.5 ± 0.4	0.3 ± 0.7	2.1 ± 3.3	**0.015**
Urine protein (mg/L)	126 ± 68	346 ± 615	285 ± 365	1357 ± 1776	**0.005**
Urine osmolarity (mOsmol/kg)	499 ± 140	405 ± 164	369 ± 51	336 ± 99	**0.011**

Values are mean ± SD or number and percentages.

AKI, acute kidney injury; HRS, hepatorenal syndrome; ATN, acute tubular necrosis; INR, international normalized ratio; MELD, model for end-stage liver disease.

### Urinary biomarkers and relationship with Acute Kidney Injury

We first analyzed the relationship between the different urinary biomarkers and the presence or absence of AKI. Median urinary concentrations of NGAL, osteopontin, MCP-1, albumin, and TFF-3 in patients with AKI were significantly higher compared to those of patients without AKI (Table 3). By contrast, there were no significant differences between patients with and without AKI with respect to the urinary concentrations of KIM-1, GST-π, cystatin C, calbindin, β2-microglobulin, IL-18, and clusterin. Remarkably, when patients were categorized according to AKI stages the urinary levels of most biomarkers were not significantly different among the different stages (Table 4). The only two biomarkers that were significantly different across AKI stages were MCP-1 and albumin.

**Table 3:** Values of urinary biomarkers according to diagnosis of AKI.

Urinary Biomarkers	No AKI (n = 16)	AKI (n = 39)	*p*
NGAL (μg/g creat)	30 (17–41)	118 (38–617)	**< 0.0001**
Osteopontin (μg/g creat)	1456 (715–3210)	5471 (2345–11983)	**< 0.0001**
MCP-1 (μg/g creat)	0.2 (0.1–1.4)	2.2 (0.7–5.3)	**0.001**
Albumin (mg/g creat)	3 (1–7)	39 (8–128)	**0.001**
TFF-3 (μg/g creat)	582 (367–1665)	2474 (840–4067)	**0.001**
KIM-1 (μg/g creat)	0.5 (0.3–1.4)	1.2 (0.4–2.6)	0.111
GST-π (μg/g creat)	3 (1–16)	6 (2–43)	0.129
Cystatin C (μg/g creat)	24 (12–45)	37 (15–135)	0.129
Calbindin (μg/g creat)	71 (26–150)	27 (6–141)	0.195
β2-microglobulin (μg/g creat)	51 (13–140)	76 (5–590)	0.447
IL-18 (ng/g creat)	21 (16–35)	29 (14–129)	0.684
Clusterin (μg/g creat)	9 (2–38)	9 (1–26)	0.926

Values are expressed as median and IQR.

AKI, acute kidney injury.

**Table 4:** Values of urinary biomarkers according to AKI stage at diagnosis of AKI.

Urinary Biomarkers	AKI-1 (n = 13)	AKI-2 (n = 15)	AKI-3 (n = 11)	*p*
NGAL (μg/g creat)	187 (31–430)	111 (58–424)	437 (92–2515)	0.259
Osteopontin (μg/g creat)	4927 (1925–15345)	4868 (2704–7280)	6436 (3501–15772)	0.499
MCP-1 (μg/g creat)	1.2 (0.3–3.3)	2.0 (0.9–2.9)	8.2 (2.5–14.7)	**0.015**
Albumin (mg/g creat)	16 (2–104)	17 (3–66)	319 (20–381)	**0.029**
TFF-3 (μg/g creat)	2474 (427–4548)	2632 (1893–4017)	2039 (553–4753)	0.670
KIM-1 (μg/g creat)	0.6 (0.1–3.5)	1.2 (0.6–1.7)	1.6 (0.3–3.7)	0.559
GST-π (μg/g creat)	4 (1–20)	8 (2–37)	8 (5–87)	0.443
Cystatin C (μg/g creat)	39 (17–777)	37 (20–77)	14 (8–212)	0.544
Calbindin (μg/g creat)	36 (4–168)	27 (6–116)	34 (9–148)	0.815
β2-microglobulin (μg/g creat)	156 (7–1553)	76 (5–239)	19 (3–1345)	0.773
IL-18 (ng/g creat)	23 (15–44)	29 (14–170)	53 (13–163)	0.916
Clusterin (μg/g creat)	9 (1–83)	13 (4–26)	4 (1–18)	0.370

Values are expressed as median and IQR.

AKI, acute kidney injury.

In sharp contrast with the lack of differences in most biomarkers among different AKI stages, when patients were classified into 4 categories according to the presence and type of AKI there were statistically significant differences in all biomarkers among the four groups, except for clusterin (Table 5). As a whole, however, there was overlap of biomarker levels between the different categories. In all biomarkers, the highest median values were found in patients with ATN and the lowest in patients without AKI or with Prerenal-AKI. Overall, in patients with type-1 HRS biomarker levels had intermediate values between those of patients with ATN and Prerenal-AKI.

**Table 5:** Values of urinary biomarkers in patients categorized according to the absence or presence of AKI and type of AKI.

Urinary Biomarkers	No AKI(n = 16)	Prerenal (n = 12)	HRS Type I (n = 15)	ATN(n = 12)	*p*
NGAL (μg/g creat)	30 (17–41)	36 (26–125)	104 (58–208)	1807 (494–3716)	**< 0.0001**
IL-18 (ng/g creat)	21 (16–35)	16 (14–36)	18 (10–29)	150 (58–259)	**< 0.0001**
Albumin (mg/g creat)	3 (1–7)	9 (1–77)	16 (8–46)	324 (53–380)	**< 0.0001**
TFF-3 (μg/g creat)	582 (367–1665)	2300 (323–2720)	1893 (840–2715)	5810 (2778–13332)	**< 0.0001**
MCP-1 (μg/g creat)	0.2 (0.1–1.4)	0.9 (0.2–2.5)	3 (1–6)	4 (1–14)	**< 0.0001**
Osteopontin (μg/g creat)	1456 (715–3210)	2914 (1847–8382)	5471 (2959–11983)	8337 (4019–14466)	**< 0.0001**
Calbindin (μg/g creat)	71 (26–150)	5 (2–34)	25 (8–58)	118 (37–324)	**0.010**
GST-π (μg/g creat)	3 (1–16)	3 (1–7)	4 (2–21)	50 (9–169)	**0.012**
KIM-1 (μg/g creat)	0.5 (0.3–1.4)	0.5 (0.1–1.1)	1.2 (0.5–2.8)	1.7 (0.9–5.1)	**0.015**
Cystatin C (μg/g creat)	24 (12–45)	21 (15–153)	27 (10–47)	115 (39–1552)	**0.023**
β2-microglobulin (μg/g creat)	51 (13–140)	50 (4–336)	19 (3–239)	1094 (41–26709)	**0.033**
Clusterin (μg/g creat)	9 (2–38)	1 (0.9–12)	7 (2–26)	20 (6–255)	0.103

Values are expressed as median and IQR.

AKI, acute kidney injury; HRS, hepatorenal syndrome; ATN, acute tubular necrosis.

Values in healthy subjects are as follows: NGAL 33 (23–47), IL-18 18 (11–45), Albumin 1 (0–2), TFF-3 678 (466–951), MCP-1 0.1 (0.04–0.2), Osteopontin 1416 (900–2025), Calbindin 20 (5–58), GST-π 0.9 (0.3–5.9), KIM-1 0.2 (0.1–0.4), Cystatin C 32 (8–57), β2-microglobulin 95 (9–132), Clusterin 10 (2–79).

The differential diagnosis between ATN and the other two types of AKI is often difficult in clinical practice but is very important in guiding therapeutic interventions. Therefore, we next assessed the ability of biomarkers in the differential diagnosis of ATN from other types of AKI. The predictive accuracy of each of the biomarkers to differentiate ATN from the other two types of AKI was calculated. As shown in Table 6, the biomarker with the highest predictive accuracy for ATN diagnosis was NGAL with an AUCROC of 0.957. The best cut-off level of NGAL to differentiate ATN from other types of AKI was of 294 μg/g creatinine. Eleven out of the 12 patients with ATN had a urinary NGAL value above this threshold. Biomarkers with statistically significant AUCROC for ATN diagnosis above 0.80 were IL-18, albumin, TFF-3, and GST-π.

**Table 6:** AUCROC of all biomarkers for the differential diagnosis of ATN vs other types of AKI.

Urinary Biomarkers	Diagnosis of ATN vs other types of AKIAUCROC (95% CI)	*p*	Cut-off level	Sensitivity	Specificity
NGAL (μg/g creat)	0.957 (0.891–1.00)	**< 0.0001**	294	92%	89%
IL-18 (ng/g creat)	0.920 (0.836–1.00)	**< 0.0001**	51	83%	89%
Albumin (mg/g creat)	0.858 (0.733–0.983)	**< 0.0001**	86	75%	85%
TFF-3 (μg/g creat)	0.824 (0.667–0.981)	**0.001**	3040	75%	81%
GST-π (μg/g creat)	0.812 (0.647–0.976)	**0.002**	8.27	83%	78%
β2-microglobulin (μg/g creat)	0.790 (0.624–0.956)	**0.004**	410	67%	85%
Calbindin (μg/g creat)	0.784 (0.638–0.930)	**0.005**	41.4	75%	74%
Cystatin C (μg/g creat)	0.762 (0.594–0.931)	**0.010**	44.5	75%	70%
Clusterin (μg/g creat)	0.704 (0.502–0.906)	**0.045**	16.2	67%	78%
KIM-1 (μg/g creat)	0.704 (0.518–0.889)	**0.045**	1.6	67%	74%
MCP-1 (μg/g creat)	0.685 (0.490–0.881)	0.068	2.2	67%	59%
Osteopontin (μg/g creat)	0.639 (0.442–0.836)	0.171	5680	58%	59%

ATN, acute tubular necrosis; AKI, acute kidney injury.

### Biomarkers and relationship with ACLF

We then analyzed the relationship between the different urinary biomarkers and the presence or absence of ACLF. Median urinary concentrations of NGAL, osteopontin, albumin, MCP-1, TFF-3, KIM-1, and GST-π in patients with ACLF were significantly higher compared to those of patients without ACLF (Table 7). Moreover, NGAL, osteopontin, albumin, MCP-1, and TFF-3 were very accurate in ACLF diagnosis with an AUCROC greater than 0.75. By contrast, there were no significant differences between patients with and without ACLF with respect to the urinary concentrations of cystatin C, clusterin, IL-18, β2-microglobulin, and calbindin.

**Table 7:** Values of urinary biomarkers in patients categorized according to the absence or presence of Acute-on-chronic liver failure (ACLF).

Urinary Biomarkers	No ACLF (n = 21)	ACLF (n = 34)	*p*	Diagnosis of ACLF AUCROC (95% CI)
NGAL (μg/g creat)	32 (19–40)	165 (67–676)	**< 0.0001**	0.878 (0.787–0.969)
Osteopontin (μg/g creat)	1669 (820–2987)	5560 (3365–11028)	**< 0.0001**	0.839 (0.715–0.963)
Albumin (mg/g creat)	2 (1–7)	41 (11–281)	**< 0.0001**	0.829 (0.718–0.940)
MCP-1 (μg/g creat)	0.2 (0.1–1.4)	2.2 (1–6.3)	**< 0.0001**	0.812 (0.691–0.934)
TFF-3 (μg/g creat)	649 (368–1932)	2574 (902–4239)	**0.001**	0.768 (0.641–0.894)
KIM-1 (μg/g creat)	0.4 (0.1–1)	1.3 (0.6–3.4)	**0.002**	0.746 (0.614–0.879)
GST-π (μg/g creat)	2 (1–6)	8 (3–47)	**0.008**	0.713 (0.571–0.854)
Cystatin C (μg/g creat)	21 (15–43)	40 (13–184)	0.072	0.646 (0.502–0.789)
Clusterin (μg/g creat)	3 (1–14)	13 (3–40)	0.074	0.644 (0.493–0.795)
IL-18 (ng/g creat)	23 (16–34)	31 (14–165)	0.299	0.584 (0.433–0.735)
β2-microglobulin (μg/g creat)	52 (11–149)	90 (5–756)	0.341	0.577 (0.426–0.728)
Calbindin (μg/g creat)	49 (6–149)	32 (9–143)	0.876	0.487 (0.328–0.647)

Values are expressed as median and IQR.

ACLF, Acute-on-chronic liver failure

Due to the design of the study which was based on selection of patients according to presence or absence of AKI, the vast majority of patients with ACLF had associated AKI (33 out of 34). Therefore, the possibility existed that the increased urinary levels of some of the biomarkers could be explained largely by the associated AKI. To further explore this issue we studied an additional group of patients with ACLF but without associated AKI. The characteristics of this group of patients are shown in Table B in S1 File. We then compared urinary biomarkers in all patients of the study categorized in 4 groups: patients without ACLF with or without AKI, and patients with ACLF with or without AKI. As shown in Table 8, urinary biomarkers associated with the presence of ACLF were NGAL, osteopontin, albumin, and TFF3. By contrast, MCP-1, KIM-1, and GST-π were only significantly increased in patients with ACLF in the setting of associated AKI, which suggests that these biomarkers were likely related to kidney injury.

**Table 8:** Values of urinary biomarkers in patients categorized according to the absence or presence of Acute-on-chronic liver failure (ACLF) and Acute kidney injury (AKI).

Urinary Biomarkers	No ACLF without AKI (n = 15)	No ACLF with AKI (n = 6)	ACLF without AKI (n = 11)	ACLF with AKI (n = 33)	*p*
NGAL (μg/g creat)	32 (16–43)	31 (25–89)	147 (41–233)	187 (75–688)	**< 0.0001**
Osteopontin (μg/g creat)	1444 (664–2541)	1970 (1445–17478)	3990 (2033–5662)	5650 (3230–11346)	**< 0.0001**
Albumin (mg/g creat)	1.8 (0.8–6.6)	1.8 (1.2–32)	50 (24–118)	42 (10–293)	**< 0.0001**
TFF-3 (μg/g creat)	649 (338–1708)	1383 (369–3113)	3117 (1327–3856)	2611 (1052–4410)	**0.002**
MCP-1 (μg/g creat)	0.1 (0.1–1.2)	0.7 (0.1–2.9)	0.8 (0.2–2.3)	2.3 (1–6.9)	**0.001**
KIM-1 (μg/g creat)	0.4 (0.3–1.4)	0.3 (0.1–0.9)	0.7 (0.3–1.2)	1.3 (0.6–3.0)	**0.016**
GST-π (μg/g creat)	2.4 (0.6–6.4)	1.4 (0.8–41)	3.2 (2.8–53)	7.6 (3.2–43)	0.069
Clusterin (μg/g creat)	8 (1.7–33)	1.1 (0.8–3.2)	16 (0.3–39)	13 (2.8–35)	0.097
Cystatin C (μg/g creat)	21 (11–47)	21 (15–51)	34 (20–44)	42 (13–193)	0.308
Calbindin (μg/g creat)	58 (26–151)	5 (4–1216)	58 (29–154)	31 (8–144)	0.366
IL-18 (ng/g creat)	19 (16–30)	27 (15–39)	34 (26–196)	29 (14–150)	0.368
β2-microglobulin (μg/g creat)	52 (13–144)	50 (7–215)	121 (43–218)	103 (5–785)	0.598

Values are expressed as median and IQR.

ACLF, Acute-on-chronic liver failure; AKI, acute kidney injury.

### Biomarkers and relationship with survival

Finally, a number of biomarkers were related to the occurrence of death within a 3 month period (Table 9). The biomarkers with the highest AUCROC of mortality were NGAL, osteopontin, albumin, and MCP-1.

**Table 9:** Values of urinary biomarkers in patients categorized according to survival at 3 months*.

Urinary Biomarkers	Alive (n = 37)	Dead (n = 14)	*p*	Mortality AUCROC (95% CI)
NGAL (μg/g creat)	38 (27–108)	458 (114–3661)	**< 0.0001**	0.876 (0.782–0.971)
Osteopontin (μg/g creat)	2704 (1442–5391)	8759 (4770–16455)	**0.001**	0.809 (0.683–0.935)
Albumin (mg/g creat)	7 (2–37)	113 (15–333)	**0.001**	0.797 (0.656–0.938)
MCP-1 (μg/g creat)	1.1 (0.1–2.4)	3.4 (2.0–9.3)	**0.001**	0.795 (0.665–0.925)
GST-π (μg/g creat)	4 (1–15)	26 (5–58)	**0.010**	0.736 (0.599–0.872)
KIM-1 (μg/g creat)	0.6 (0.3–1.6)	2.1 (0.6–5.4)	**0.021**	0.710 (0.540–0.881)
TFF-3 (μg/g creat)	1538 (483–2673)	3208 (899–8700)	**0.045**	0.683 (0.506–0.860)
Cystatin C (μg/g creat)	26 (12–47)	57 (20–344)	0.095	0.653 (0.482–0.823)
IL-18 (ng/g creat)	18 (15–39)	62 (20–180)	0.100	0.651 (0.450–0.851)
β2-microglobulin (μg/g creat)	60 (6–211)	73 (15–7793)	0.410	0.575 (0.389–0.761)
Clusterin (μg/g creat)	9 (1–30)	11 (3–111)	0.423	0.573 (0.388–0.758)
Calbindin (μg/g creat)	49 (8–129)	27 (9–244)	1	0.500 (0.318–0.682)

Values are expressed as median and IQR.

*Three patients were lost to follow-up and one patient was transplanted during the 3-month follow-up period.

## Discussion

The current study was designed to evaluate the potential role of a panel of urinary biomarkers in the assessment of clinical outcomes in patients with cirrhosis. The novelty of this study with respect to previous studies published examining urinary biomarkers in patients with cirrhosis is twofold [11–15]. First, the analytical approach used was an analysis of a panel of biomarkers instead of just one or few biomarkers. This was possible with the use of the multiplex technology, which allows testing for a significant number of biomarkers using a small urine volume. Second, the current study analyzed not only the potential usefulness of biomarkers in the assessment of AKI, but also its possible effectiveness in the assessment of the recently described syndrome of ACLF and survival.

The first outcome analyzed was the presence, severity, and type of AKI. Urinary biomarkers reflect typically, but not exclusively, alterations in kidney function such as impairment of glomerular filtration rate and/or tubular injury or dysfunction [6,7]. Not unexpectedly, some biomarkers were found to be increased in patients with AKI compared to those of patients without AKI, including NGAL and albumin. NGAL has previously been reported as increased in the setting of AKI in cirrhosis [11–15]. Interestingly, the urinary levels of some biomarkers not evaluated in previous studies, such as TFF-3, MCP-1, and osteopontin were higher in patients with AKI compared to those of patients without AKI.

In the clinical setting, the diagnosis of AKI is made on the basis of changes in serum creatinine concentration according to pre-established criteria [17]. Therefore, there is no need for the use of biomarkers to diagnose AKI. By contrast, the usefulness of biomarkers in the field of cirrhosis may be particularly relevant in the differential diagnosis of the type of AKI, especially in distinguishing ATN from Prerenal-AKI or HRS. Theoretically, the urinary concentration of biomarkers which are up-regulated in the setting of tubular injury should be increased in patients with ATN compared to patients with Prerenal-AKI. Moreover, HRS should also theoretically display low levels of biomarkers in urine because of the anticipated lack of injury in the tubular cells [22,23]. Therefore, we analyzed the predictive value of all biomarkers included in the panel in the differential diagnosis between ATN and Prerenal-AKI or type-1 HRS. The majority of biomarkers analyzed had statistically significant predictive value in the diagnosis of ATN, except for MCP-1 and osteopontin. The biomarkers with the highest predictive value were NGAL, IL-18, albumin, TFF-3, and GST-π. The NGAL findings are consistent with those of a previous study from our group as well as from other groups [11–13]. The level of NGAL with the highest sensitivity and specificity for the diagnosis of ATN found in the current study was 294 μg/g creatinine. Taken together, the findings of these studies suggest that uNGAL is of value in the differential diagnosis of ATN from functional types of AKI, including Prerenal-AKI and HRS. Future studies should therefore aim at evaluating the incorporation of uNGAL determination in the algorithm of differential diagnosis of AKI in cirrhosis. This biomarker-based algorithm could be useful in guiding therapeutic decisions in these patients. Biomarkers other than NGAL that showed high accuracy (AUCROC > 0.8) in ATN prediction were IL-18, albumin, TFF-3, and GST-π. The first two biomarkers have also been reported to have high sensitivity and specificity in ATN diagnosis in a recent study [13]. The possible role of TFF-3 and GST-π in ATN diagnosis has to our knowledge not been reported previously and would require confirmation in future studies. TFF-3 is a member of the trefoil factor peptide family that in the kidney it is predominantly produced by epithelial cells of the proximal and distal tubules and cortical collecting duct that appears to play a role in tubular regeneration early after injury [24]. Information on the potential usefulness of TFF-3 as urinary biomarker of kidney injury is very scant [25]. Interestingly, a recent study has shown that the plasma levels of TFF-3 are increased in patients with AKI before liver transplantation and return to normal values following recovery of kidney function after transplantation [26]. GST-π belongs to the family of glutathione-S-transferases that are ubiquitous enzymes that act by detoxifying free radicals. They are present in a large variety of tissues, but the α and π isoforms are very abundant in kidney tubules and are overexpressed after kidney injury [27]. Evidence indicates that urinary GST-π levels are helpful for early diagnosis and predicting progression of ATN after cardiac surgery [28]. On the other hand, the potential usefulness of urinary KIM-1 in the diagnosis of ATN that has been reported in nephrology studies in non-selected patient population was not confirmed in the current study in patients with cirrhosis, as this biomarker had one of the lowest AUCROC curves of all biomarkers analyzed. With the results of the current study, however, it is not known whether the increase in urinary biomarkers in patients with cirrhosis and ATN was exclusively due to ATN or could, at least in part, be related to the concomitant presence of cirrhosis. Unfortunately, a group of patients with ATN without cirrhosis was not available for study.

It is interesting to emphasize that while the urinary biomarkers reported above were helpful in differentiating the type of AKI (ATN vs other), there were no significant differences in biomarker levels according to AKI stage (Table 4). This suggests that in cirrhosis, urinary biomarker concentrations are more dependent on the etiology than on the severity of AKI, as assessed by AKI stage.

An interesting and novel finding of the current study was that some of the urinary biomarkers studied were increased in patients with ACLF compared to levels in patients with acute decompensation of cirrhosis without ACLF, and showed high predictive accuracy of ACLF diagnosis. ACLF is a syndrome recently described that occurs in patients with an acute decompensation of cirrhosis and is characterized by the presence of failure of different organs/systems associated with high short-term mortality rate [4]. Although the pathogenesis of this syndrome is not fully understood, most patients with ACLF show signs of systemic inflammatory response which is thought to play a role in the development of organ failures. In some patients the inflammatory response is related to bacterial infections while in others it occurs in the absence of any detectable infection [29–31]. Potential useful biomarkers for ACLF included NGAL, osteopontin, albumin, MCP-1, KIM-1, and TFF-3. Interestingly, these biomarkers were also highly predictive of 3-month survival. The value of NGAL as biomarker of ACLF is consistent with the results found in a large population of patients from the Canonic study showing that uNGAL levels correlated with the presence and severity of ACLF and showed independent predictive value of short-term survival [32]. Moreover, preliminary studies from our laboratory show that the lipocalin-2 gene expression, the gene responsible for NGAL synthesis, is markedly upregulated in the liver of patients with ACLF compared to the expression found in the liver of patients with acute decompensation of cirrhosis without ACLF (unpublished observations). These findings are in keeping with those of several recent experimental studies showing up-regulation of the lipocalin-2 gene following hepatocyte injury, bacterial infection or during liver regeneration [8–10]. Taken together, these results suggest that the increased NGAL levels in the urine of patients with ACLF may derive not only from an injured kidney but also from the liver. Moreover, there is the possibility, not yet tested, of the contribution of leukocytes to increased uNGAL levels, because gene expression may also be upregulated as a consequence of leukocyte activation. The association between MCP-1 and KIM-1 and ACLF was related to the existence of associated AKI. It is well known that KIM-1 is overexpressed in the kidney as a consequence of different types of injury and its urinary levels are increased in this condition [7]. Likewise, MCP-1 expression is increased in the kidney in experimental models of AKI [33]. Therefore, the possibility exists that MCP-1 found in urine in patients with ACLF may be in part from renal origin.

The potential role of osteopontin and TFF-3 as biomarkers of ACLF deserves a specific comment. Osteopontin is a pleotropic protein that is expressed in a number of cell types, including osteoblasts, chondrocytes, inflammatory cells (macrophages, dendritic cells and T-cells), kidney cells, hepatocytes, and hepatic stellate cells. Osteopontin secreted from the cells acts through several of receptors, including various integrins and CD44, regulating a number of cellular processes such as bone mineralization, tissue remodeling, and inflammation [34]. Several lines of evidence indicate that osteopontin plays a role in conditions characterized by chronic inflammatory state. In the liver, increased osteopontin expression has been found in several experimental models of liver diseases as well as in human diseases, particularly alcoholic hepatitis and NASH [35,36]. Osteopontin has proinflammatory and profibrogenic properties in the liver. Investigations from our laboratory indicate that osteopontin is overexpressed in the liver of patients with alcoholic hepatitis and its expression correlates with inflammatory activity within the liver and disease severity [35]. Similar findings have been found in patients with non-alcoholic steatohepatitis [36]. On this background, the findings of the current study of increased urinary osteopontin levels in patients with ACLF are consistent with the existence of a marked inflammatory reaction in this condition [4]. Whether the high urinary levels are secondary to an increased systemic production of osteopontin, either derived from the liver and/or from inflammatory cells, or reflect a theoretically increased expression within the kidneys or a combination thereof is not known. The potential role of osteopontin as biomarker of ACLF should be explored in future studies.

Several lines of evidence indicate that TFF-3 is a protective factor in the intestinal epithelium and plays a beneficial role in inflammatory bowel diseases in humans [37]. TFF-3 is also expressed in the biliary epithelium and its expression increases in conditions of injury of biliary epithelial cells [38,39]. The origin of the increased urine levels of TFF-3 in patients with ACLF found in the current study is unknown. Nevertheless, the potential role of TFF-3 as biomarker in this condition deserves investigation in future studies. A final interesting observation of the current study is the markedly increased urinary levels of albumin in patients with ACLF both with and without AKI. Previous studies have shown that urine albumin could be a potential biomarker of AKI in patients with cirrhosis [13]. Our study extends these observations by showing that patients with ACLF have increased urine albumin levels regardless of the presence of AKI. This suggests a potential role for urine albumin as biomarker of ACLF. Moreover, urine albumin was also associated with prognosis. These findings deserve investigation in larger series of patients.

In summary, in the current study we found an association of several urinary biomarkers with different outcomes in patients with cirrhosis. A number of biomarkers were associated with the presence of ATN and were useful in distinguishing ATN from other causes of AKI. The best biomarkers in the differential diagnosis of ATN were NGAL, IL-18, albumin, TFF-3, and GST-π. On the other hand, we found a number of biomarkers associated with ACLF, including among others NGAL, osteopontin, albumin, and TFF-3. The increased levels of NGAL, osteopontin, and TFF-3 point towards the importance of inflammatory mediators as biomarkers in this condition. Finally, the same four biomarkers were associated with 3-month survival. The application of these biomarkers in clinical practice and its potential usefulness in assessing outcomes in patients with cirrhosis should be evaluated in prospective studies including large patient populations.

## Supporting Information

S1 File Table A, Comparison of baseline characteristics between cirrhotic patients with and without AKI. Table B, Demographic, clinical data and liver and kidney function of patients with ACLF and without associated AKI.
(DOC)

## References

[1] Cárdenas A, Solà E, Graupera I, Ginès P. Cirrhosis and its complications In: ACP Medicine. American College of Physicians 6 2013.

[2] Schuppan D, Afdhal NH. Liver cirrhosis. Lancet 2008;371:838–851. 10.1016/S0140-6736(08)60383-9 18328931 PMC2271178

[3] Jalan R, Ginès P, Olson JC, Mookerjee RP, Moreau R, Garcia-Tsao G, et al Acute-on chronic liver failure. J Hepatol 2012;57:1336–1348. 10.1016/j.jhep.2012.06.026 22750750

[4] Moreau R, Jalan R, Ginès P, Pavesi M, Angeli P, Cordoba J, et al for the CANONIC Study Group. Acute-on-chronic liver failure is a distinct syndrome that develops in patients with acute decompensation of cirrhosis. Gastroenterology 2013;144:1426–1437. 10.1053/j.gastro.2013.02.042 23474284

[5] Kamath PS, Kim WR, Advanced Liver Disease Study Group. The model for end-stage liver disease (MELD). Hepatology 2007;45:797–805. 17326206 10.1002/hep.21563

[6] Koyner JL, Parikh CR. Clinical utility of biomarkers of AKI in cardiac surgery and critical illness. Clin J Am Soc Nephrol 2013;8:1034–1042. 10.2215/CJN.05150512 23471130

[7] Murray PT, Mehta RL, Shaw A, Ronco C, Endre Z, Kellum JA, et al Potential use of biomarkers in acute kidney injury: report and summary of recommendations from the 10th Acute Dialysis Quality Initiative consensus conference. Kidney Int 2014;85:513–521. 10.1038/ki.2013.374 24107851 PMC4198530

[8] Borkham-Kamphorst E, Drews F, Weiskirchen R. Induction of lipocalin-2 expression in acute and chronic experimental liver injury moderated by pro-inflammatory cytokines interleukin-1β through nuclear factor-κB activation. Liver Int 2011;31:656–665. 10.1111/j.1478-3231.2011.02495.x 21457438

[9] Borkham-Kamphorst E, van de Leur E, Zimmermann HW, Karlmark KR, Tihaa L, Haas U, et al Protective effects of lipocalin-2 (LCN2) in acute liver injury suggest a novel function in liver homeostasis. Biochim Biophys Acta 2013;1832:660–673. 10.1016/j.bbadis.2013.01.014 23376114

[10] Xu MJ, Feng D, Wu H, Wang H, Chan Y, Kolls J, et al The liver is the major source of elevated serum lipocalin-2 levels after bacterial infection or partial hepatectomy: A critical role for IL-6/STAT3. Hepatology 2015;61:692–702. 10.1002/hep.27447 25234944 PMC4303493

[11] Fagundes C, Pepin MN, Guevara M, Barreto R, Casals G, Solà E, et al Urinary neutrophil gelatinase-associated lipocalin as biomarker in the differential diagnosis of impairment of kidney function in cirrhosis. J Hepatol 2012;57:267–273. 10.1016/j.jhep.2012.03.015 22521351

[12] Verna EC, Brown RS, Farrand E, Pichardo EM, Forster CS, Sola-Del Valle DA, et al Urinary neutrophil gelatinase-associated lipocalin predicts mortality and identifies acute kidney injury in cirrhosis. Dig Dis Sci 2012;57:2362–2370. 10.1007/s10620-012-2180-x 22562534 PMC3979299

[13] Belcher JM, Sanyal AJ, Peixoto AJ, Perazella MA, Lim J, Thiessen-Philbrook H, et al for the TRIBE-AKI Consortium. Kidney biomarkers and differential diagnosis of patients with cirrhosis and acute kidney injury. Hepatology 2014;60:622–632. 10.1002/hep.26980 24375576 PMC4065642

[14] Belcher JM, Garcia-Tsao G, Sanyal AJ, Thiessen-Philbrook H, Peixoto AJ, Perazella MA, et al for the TRIBE-AKI Consortium. Urinary Biomarkers and Progression of AKI in Patients with Cirrhosis. Clin J Am Soc Nephrol 2014;9:1857–1867. 10.2215/CJN.09430913 25183658 PMC4220770

[15] Barreto R, Elia C, Solà E, Moreira R, Ariza X, Rodríguez E, et al Urinary neutrophil gelatinase-associated lipocalin predicts kidney outcome and death in patients with cirrhosis and bacterial infections. J Hepatol 2014;61:35–42. 10.1016/j.jhep.2014.02.023 24613364

[16] Bai JP, Bell R, Buckman S, Burckart GJ, Eichler HG, Fang KC, et al Translational biomarkers: from preclinical to clinical a report of 2009 AAPS/ACCP Biomarker Workshop. AAPS J 2011;13:274–283. 10.1208/s12248-011-9265-x 21448748 PMC3085704

[17] Kidney Disease: Improving Global Outcomes (KDIGO) Acute Kidney Injury Work Group: KDIGO clinical practice guideline for acute kidney injury. Kidney Int Suppl 2012;2:1–138.

[18] Fagundes C, Barreto R, Guevara M, Garcia E, Solà E, Rodríguez E, et al A modified acute kidney injury classification for diagnosis and risk stratification of impairment of kidney function in cirrhosis. J Hepatol 2013;59:474–481. 10.1016/j.jhep.2013.04.036 23669284

[19] Ginès P, Angeli P, Lenz K, Møller S, Moore K, Moreau R, et al EASL clinical practice guidelines on the management of ascites, spontaneous bacterial peritonitis, and hepatorenal syndrome in cirrhosis. J Hepatol 2010;53:397–417. 10.1016/j.jhep.2010.05.004 20633946

[20] Ginès P, Fernández J, Durand F, Saliba F. Management of critically-ill cirrhotic patients. J Hepatol 2012;56:S13–S24. 10.1016/S0168-8278(12)60003-8 22300462

[21] Piano S, Rosi S, Maresio G, Fasolato S, Cavallin M, Romano A, et al Evaluation of the acute kidney injury network criteria in hospitalized patients with cirrhosis and ascites. J Hepatol 2013;59:482–489. 10.1016/j.jhep.2013.03.039 23665185

[22] Ginès P, Schrier RW. Renal failure in cirrhosis. N Engl J Med 2009;361:1279–1290. 10.1056/NEJMra0809139 19776409

[23] Papper S. Hepatorenal Syndrome In: The Kidney in Liver Disease. Epstein M (ed) Elsevier Biomedical, New York;1983:87–106.

[24] Hoffmann W. Trefoil factors TFF (trefoil factor family) peptide-triggered signals promoting mucosal restitution. Cell Mol Life Sci 2005;62:2932–2938. 16374581 10.1007/s00018-005-5481-9 PMC11139160

[25] O'Seaghdha CM, Hwang SJ, Larson MG, Meigs JB, Vasan RS, Fox CS. Analysis of a urinary biomarker panel for incident kidney disease and clinical outcomes. J Am Soc Nephrol 2013;24:1880–1888. 10.1681/ASN.2013010019 23990678 PMC3810081

[26] Levitsky J, Baker TB, Jie C, Ahya S, Levin M, Friedewald J, et al Plasma protein biomarkers enhance the clinical prediction of kidney injury recovery in patients undergoing liver transplantation. Hepatology 2014;60:2017–2026. 10.1002/hep.27346 25078558

[27] McMahon BA, Koyner JL, Murray PT. Urinary glutathione S-transferases in the pathogenesis and diagnostic evaluation of acute kidney injury following cardiac surgery: a critical review. Curr Opin Crit Care 2010;16:550–555. 10.1097/MCC.0b013e32833fdd9a 20930627 PMC4363992

[28] Koyner JL, Vaidya VS, Bennett MR, Ma Q, Worcester E, Akhter SA, et al Urinary biomarkers in the clinical prognosis and early detection of acute kidney injury. Clin J Am Soc Nephrol 2010;5:2154–2165. 10.2215/CJN.00740110 20798258 PMC2994075

[29] Jalan R, Fernández J, Wiest R, Schnabl B, Moreau R, Angeli P, et al Bacterial infections in cirrhosis: a position statement based on the EASL Special Conference 2013. J Hepatol 2014;60:1310–1324. 10.1016/j.jhep.2014.01.024 24530646

[30] Jalan R, Yurdaydin C, Bajaj JS, Acharya SK, Arroyo V, Lin HC, et al; World Gastroenterology Organization Working Party. Toward an improved definition of acute-on-chronic liver failure. Gastroenterology 2014;147:4–10. 10.1053/j.gastro.2014.05.005 24853409

[31] Moreau R, Arroyo V. Acute on Chronic Liver Failure: A New Clinical Entity. Clin Gastroenterol Hepatol 2014 2 28 [Epub ahead of print]. 10.1016/j.cgh.2014.02.027 24583872

[32] Ariza X, Angeli P, Barreto R, Jalan R, Morales Ruiz M, Elia C, et al CANONIC Study Investigators of the EASL CLIF Consortium. Urinary neutrophil gelatinase-associated lipocalin (NGAL) is a strong predictor of short-term outcome in patients with acute decompensation of cirrhosis. Relationship with Acute-on-chronic liver failure (ACLF). J Hepatol 2014;60 (Suppl. 1):S30–S31.

[33] Munshi R, Johnson A, Siew ED, Ikizler TA, Ware LB, Wurfel MM, et al MCP-1 gene activation marks acute kidney injury. J Am Soc Nephrol 2011;22:165–175. 10.1681/ASN.2010060641 21071523 PMC3014045

[34] Kahles F, Findeisen HM, Bruemmer D. Osteopontin: A novel regulator at the cross roads of inflammation, obesity and diabetes. Mol Metab 2014;3:384–393. 10.1016/j.molmet.2014.03.004 24944898 PMC4060362

[35] Morales-Ibañez O, Domínguez M, Ki SH, Marcos M, Chaves JF, Nguyen-Khac E, et al Human and experimental evidence supporting a role for osteopontin in alcoholic hepatitis. Hepatology 2013;58:1742–1756. 10.1002/hep.26521 23729174 PMC3877722

[36] Syn WK, Agboola KM, Swiderska M, Michelotti GA, Liaskou E, Pang H, et al NKT-associated hedgehog and osteopontin drive fibrogenesis in non-alcoholic fatty liver disease. Gut 2012;61:1323–1329. 10.1136/gutjnl-2011-301857 22427237 PMC3578424

[37] Aamann L, Vestergaard EM, Grønbæk H. Trefoil factors in inflammatory bowel disease. World J Gastroenterol. 2014;20:3223–3230. 10.3748/wjg.v20.i12.3223 24696606 PMC3964394

[38] Srivatsa G, Giraud AS, Ulaganathan M, Yeomans ND, Dow C, Nicoll AJ. Biliary epithelial trefoil peptide expression is increased in biliary diseases. Histopathology. 2002;40:261–268. 11895492 10.1046/j.1365-2559.2002.01347.x

[39] Sasaki M, Tsuneyama K, Saito T, Kataoka H, Mollenhauer J, Poustka A, et al Site-characteristic expression and induction of trefoil factor family 1, 2 and 3 and malignant brain tumor-1 in normal and diseased intrahepatic bile ducts relates to biliary pathophysiology. Liver Int. 2004;24:29–37. 15101998 10.1111/j.1478-3231.2004.00883.x

