# Supplementary material for: Analysis of a Urinary Biomarker Panel for Clinical Outcomes Assessment in Cirrhosis
Source: PLoS One. 2015 Jun 4;10(6):e0128145. doi: 10.1371/journal.pone.0128145 (PMC4456079; doi:10.1371/journal.pone.0128145)
Supplement: S1 File — Table A, Comparison of baseline characteristics between cirrhotic patients with and without AKI. Table B, Demographic, clinical data and liver and kidney function of patients with ACLF and without associated AKI. (DOC) [file pone.0128145.s001.doc]

**Table A. Comparison of baseline characteristics between cirrhotic patients with and without AKI.**

|  | **No AKI (n=16)** | **AKI (n=39)** | ***p*** |
| --- | --- | --- | --- |
| Age, yr | 58 ± 10 | 57 ± 10 | 0.835 |
| Male | 13 (81%) | 30 (77%) | 1 |
| Chronic impairment of kidney function* | 0 (0%) | 3 (8%)* | 1 |
| Etiology of cirrhosis: Alcoholic/ Hepatitis C / Other | 6 (38%), 6 (38%), 4 (25%) | 20 (51%), 13 (33%),  6 (15%) | 0.5 |
| Norfloxacin prophylaxis | 1 (6%) | 9 (23%) | 0.250 |
| Treatment with beta-blockers | 4 (25%) | 16 (41%) | 0.359 |
| Presence of ascites | 12 (75%) | 34 (87%) | 0.422 |
| Presence of encephalopathy | 3 (19%) | 15 (39%) | 0.213 |
| Presence of bacterial infection | 8 (50%) | 20 (51%) | 0.931 |
| Presence of shock | 1 (6%) | 11 (28%) | 0.147 |
| Serum bilirubin (mg/dL) | 1.9 ± 0.9 | 8.6 ± 11.9 | **0.001** |
| Serum albumin (g/L) | 27.5 ± 5.2 | 29.6 ± 6.4 | 0.248 |
| INR | 1.5 ± 0.4 | 1.8 ± 0.6 | 0.080 |
| MELD score | 13 ± 4 | 27 ± 7 | **< 0.0001** |
| Child-Pugh score | 8.6 ± 1.5 | 9.5 ± 2.0 | 0.102 |
| ACLF** | 1 (6%) | 33 (85%) | **< 0.0001** |
| Serum creatinine (mg/dL) | 0.7 ± 0.2 | 2.9 ± 1.5 | **< 0.0001** |
| Serum sodium (mEq/L) | 135 ± 5 | 131 ± 6 | **0.030** |
| Mean arterial pressure (mmHg) | 84 ± 14 | 72 ± 11 | **0.001** |
| Blood leukocytes (x10^9^/L) | 5.9 ± 3.1 | 9.2 ± 6.2 | **0.013** |
| C-reactive protein (mg/dL) | 3.2 ± 3.3 | 4.7 ± 3.8 | 0.273 |
| Urine sodium (mEq/L) | 46 ± 43 | 25 ± 26 | 0.079 |
| FENa (%) | 0.3 ± 0.4 | 1.0 ± 2.0 | 0.207 |
| Urine protein (mg/L) | 126 ± 68 | 643 ± 1157 | **0.009** |
| Urine osmolarity (mOsmol/kg) | 499 ± 140 | 371 ± 116 | **0.002** |

Values are mean ± SD or number and percentages.

AKI, acute kidney injury; INR, international normalized ratio; MELD, model for end-stage liver disease

* Parenchymal nephropathy in 2 patients and type-2 hepatorenal in 1 patient.

** ACLF, Acute-on-chronic liver failure; ACLF grade I in 19 patients, grade II in 7 patients and grade III in 8 patients.

**Table B. Demographic, clinical data and liver and kidney function of patients with ACLF and without associated AKI (n=10).**

| Age, yr | 54 ± 13 |
| --- | --- |
| Male, n (%) | 5 (50%) |
| Chronic impairment of kidney function, n (%) | - |
| Etiology of cirrhosis, n:   Alcoholic/hepatitis C/other | 4/3/3 |
| Norfloxacin prophylaxis | - |
| Treatment with beta-blockers | 2 (20%) |
| Presence of ascites | 10 (100%) |
| Presence of encephalopathy | 8 (80%) |
| Presence of bacterial infection | 9 (90%) |
| Presence of shock | 6 (60%) |
| Serum bilirubin (mg/dL) | 14.2 ± 5.2 |
| Serum albumin (g/L) | 29.6 ± 3.9 |
| INR | 2.7 ± 1.1 |
| MELD score | 27 ± 5 |
| Child-Pugh score | 11.6 ± 1.2 |
| ACLF* | 10 (100%) |
| Serum creatinine (mg/dL) | 0.9 ± 0.3 |
| Serum sodium (mEq/L) | 133 ± 6 |
| Mean arterial pressure (mmHg) | 80 ± 14 |
| Blood leukocytes (10^9^/L) | 10.1 ± 4.6 |
| C-reactive protein (mg/dL) | 3.9 ± 2.9 |

This is supplementary table 2 legend and footnotes:

Values are mean ± SD or number and percentages.

AKI, acute kidney injury; INR, international normalized ratio; MELD, model for end-stage liver disease.

*ACLF, Acute-on-chronic liver failure, ACLF grade I in 1 patient, grade II in 4 patients and grade III in 5 patients.
